# Phosphorus, calcium, and magnesium contents of pasture and their effect on body condition scores and body mass of communal cattle depending on natural pasture of Mogosane Village, of the North-West Province, South Africa

**DOI:** 10.1007/s11250-019-01908-z

**Published:** 2019-06-04

**Authors:** B. G. Mokolopi

**Affiliations:** Department of Agriculture and Animal Health, College of Agriculture and Environmental Sciences, University of South Africa, Science Campus, Private Bag X6, Florida, Johannesburg, 1710 South Africa

**Keywords:** Pasture minerals, Communal cattle, Natural pasture, Body condition score, Body weight

## Abstract

This research was conducted to determine the effects of phosphorus (P), calcium (Ca), and magnesium (Mg) content in pasture with body weight and body condition scores in cattle depending entirely on natural grazing. The work was done in 2006 in Mogosane Village of North-West Province, South Africa, and it was conducted from March 2006 to March 2007, with the annual rainfall of 384.38 mm maximum. A total of 25 growing mixed breed cattle aged between 6 months and 2 years old were selected randomly from a herd feeding exclusively on communal grazing. Animals were depending on natural pasture, with no supplements given. Records of body mass (BM) and body condition score (BCS) were recorded from a diverse range of animals in order to include as many different body masses and body condition scores as possible. Mean BM and BCS values are reported but there were no significant (*P* > 0.05) differences between months. Possible reasons are given in the “Results and Discussion” section. The focus of the research reported here was on the changes in the P, Ca, and Mg concentrations of pasture. Pasture samples were collected once a month for analysis to determine the contents of P, Ca, and Mg. Mineral concentrations in the pasture increased significantly (*P* < 0.05) with rainfall in January 2007, February 2007, and March 2007, and in January 2007 and February 2007, the mean values of BCS (3.90) and BM (444.05 kg) increased. After the good rains in March 2006 and April 2006, there were significant (*P* < 0.05) decreases in grass P and Mg in the following dry months along with decreases in BCS and BM. There were subsequent significant (*P* < 0.05) increases in grass P and Mg following rains in August 2006 through January 2007. Grass Ca followed a much different pattern compared with that of P and Mg with significant (*P* < 0.05) increases after periods of little or no rainfall. Mineral concentrations (P, Ca, and Mg) of grass in this research were significantly (*P* < 0.05) influenced by the rainfall making it imperative that mineral supplementation be given to animals in the dry periods.

## Introduction

Livestock in arid areas of Africa utilize crop residues which have low nutritive value, and natural pasture which depends on fluctuating rainfall (Alshafei et al. 2016). Poor nutrition especially during the dry season affects productivity of livestock and results in economic losses to the livestock owners, and according to Solomon and Mlambo (2017), livestock, particularly cattle and goats, play an important socioeconomic role in South Africa. Ruminant animal production is a means of sustainability to people living in rural, peri-urban, and urban areas (Ogunbosoye et al. 2015).

Minerals are dietary essentials for optimal growth, physiologic functions, and productivity in animals (Herdt and Hoff 2011) and are essential for maintaining livestock growth, reproduction, and health (Jones and Tracy 2013). The performance and health of grazing ruminants are dependent on the adequacy and availability of essential mineral elements from pastures and soil (Islam et al. 2003), and according to Gao et al. (2016), herbage minerals affect the performance of grazing cattle. In a given pasture, mineral levels vary from species to species, and mineral imbalances occur in different areas of the world (Fardous et al. 2010). Therefore, mineral deficiencies may affect the production of grazing livestock at pasture in most of the regions of the world, which include those of the major elements Ca, P, Mg, Na, and S, and the trace elements Co, Cu, I, Mn, Se, and Zn (Goswami et al. 2005) and can compromise cattle health and, consequently, the income to the farmers (Dermauw et al. 2013). Although hand-plucked forage pasture samples are not representative of what an animal actually grazes, they can serve as a vital diagnostic aid when sample collection by esophageal fistula is not convenient. Undoubtedly, forage analysis is a potential indicator of mineral status for ruminants as compared with that of soil (Khan et al. 2009).

Most animal and dairy scientists acknowledge successfully manipulating body condition scores (BCS) as an important management factor, influencing animal health, milk production, and reproduction in the modern dairy cow (Buckley et al. 2003). They are subjective, visual, or tactile evaluations of the amount of subcutaneous fat on a cow (Waltner et al. 1993). According to Roche et al. (2004), managing body reserves is critical for successful cow management and requires an accurate assessment of a cow’s condition, and according to them, body mass (BM) alone was not a good indicator of body reserves, as cows of a specific weight may be tall and thin or short and fat. Furthermore, scoring body condition and assessing changes in the body condition of dairy cattle have become strategic tools in both farm management and research. Changes in BCS as measured over several weeks provide useful information about the cow’s current nutrient intake relative to its requirements, and allow feeding decisions to be made effectively (Roche et al. 2007). Low BCS is used as an indirect indicator of energy balance status (Berry et al. 2007), and the public perception is that thin cows are welfare-compromised. However, no comprehensive studies on the mineral status of natural pastures on BCS and BW of cattle in communal grazing of Mogosane village of the North-West Province have been conducted. Furthermore, an understanding of how BCS and BM change with time and the content of minerals the animals consumed, and how they influence production and health, could potentially help communal farmers to take care of their animals, by at least supplementing them especially during dry periods when mineral status of the pasture is limiting. The objective of the study was, therefore, to monitor changes in grass P, Ca, and Mg related to season and rainfall, and to monitor BCS and BM of cattle subjected to natural grazing. Little is known about how pasture P, Ca, and Mg contents are affected by rainfall and how they affect BCS and BM of cattle depending only on natural grazing.

I therefore hypothesized the following:

i. Decreased rainfall will decrease P, Ca, and Mg contents of grass in pastures.
ii. Increased rainfall will increase P, Ca, and Mg contents of grass in pastures.
iii. Pasture with more P, Ca, and Mg contents will increase BCS and BM measured.
iv. Pasture with less P, Ca, and Mg contents will lower the levels BCS and BM recorded.

## Materials and methods

### Experimental procedures

This research was a part of the research project carried out for a Ph.D. degree by the senior author awarded in 2009 by North-West University, Mafikeng Campus. The work was done in 2006 in Mogosane Village in the Molopo District of North-West Province, South Africa, and it was conducted from March 2006 to March 2007, with an average annual rainfall of 384.38 mm.

Twenty-five mix breed cattle between the ages of 6 months and 2 years were randomly selected from among a group of animals feeding exclusively on communal grazing in Mogosane Village in the Molopo District of North-West Province and were used to determine how pasture P, Ca, and Mg contents affect BCS and BM of cattle depending only on natural grazing. During this project, the animals were allowed to graze only on natural pastures; no supplements were given. Pasture samples were directly collected from the veld once a month for 12 months as described by Mokolopi and Beighle (2006). They were cut using a stainless-steel knife and placed in clean cloth bags at the site (Khan et al. 2006).

Animals were ear-tagged. Records of body mass (BM) and body condition score (BCS) were also recorded with the assistance of two experienced evaluators, using 1–5 scale, as described by Wildman et al. (1982) that the method of BCS is based on a visual and tactile appraisal of body fat reserves in the back and pelvic regions. All the samples were collected once a month for a complete year to include the summer, autumn, winter, and spring seasons. Rainfall data was provided by the Department of Geography, North-West University, for the whole survey period.

### Laboratory procedures

All laboratory equipment used in the digestion and analysis of grass samples were soaked in 36% hydrochloric acid (HCl) overnight. They were then rinsed with distilled water 3 times and were dried in a hot air oven for 16 h at 106 °C. After drying, crucibles were allowed to cool in a desiccator for 6 h when they were then weighed to determine the empty weight. Pasture samples were then digested as described by Beighle et al. (1990), were analyzed for P through the FASPac II Version R2MI Auto-Analyzer (Astoria Pacific International 1992–2005), and were analyzed according to the method of Fiske and Subarrow (1925). Pasture samples were analyzed for Ca and Mg through an Atomic Absorption Spectrometer (The Analyst 700 model, 110 Bridgeport Avenue Shelton, CT 06484-4794, USA) and were analyzed as described by Trudeau and Freier (1967).

### Statistical analysis

The effect of P, Ca, and Mg contents of pasture on BM and BCS of communal cattle depending on natural pasture was analyzed using general linear model (GLM) procedures of the statistical analyses system (SAS 2010). The statistical model used was as follows:





where

*Y*_ijk_: the overall observation (BM and BCS)
*ì*: population means
*T*_1_: Effect of pasture minerals (P, Ca, and Mg)
“_ijk_: Residual effect

Where there was a significant *t* test (*P* < 0.05), Duncan multiple range was used to test the significance of differences between means.

## Results and discussion

There were significant (*P* < 0.05) decreases in grass P in the dry period, June 2006 to August 2006 (0.34 mg/g in June 2006, 0.33 mg/g in July 2006, and 0.35 mg/g in August 2006) compared with March 2006 (2.4 mg/g dry weight), April (1.74 mg/g dry weight), and May (1.17 mg/g dry weight) (Table 1) following the rainfall of 111.2 mm in March 2006 and 93.6 mm in April 2006. It is worth noting that even a moderate rainfall of 19.3 mm in August 2006 was responsible for a significant (*P* < 0.05) increase in grass P in September (1.35 mg/g dry weight) and October (1.01 mg/g dry weight) compared with 0.34 mg/g in June 2006, 0.33 mg/g in July 2006, and 0.35 mg/g in August 2006 (Table 1). These results indicate that the P content in grass can increase significantly (*P* < 0.05) in response to moderate rainfall. With only 19.3 mm of rainfall in August 2006, the P concentration in grass increased from 0.35 mg/g dry weight in August 2006 to 1.35 mg/g dry weight in September 2006, and again with moderate rainfall of 1 mm in September, 14.1 mm in Oct 2006, 33.8 mm in November 2006, 60.8 mm in January 2007, and 9.7 mm in February 2007, the P content of the grass in September, October, November, January, February, and March remained significantly (*P* < 0.05) above that of the dry period of June 2006, July 2006, and August 2006.

**Table 1:** Mean grass P, Ca, and Mg concentrations (mg/g, dry weight), mean BCS and BM (kg), and their respective SEMs and rainfall (mm) by months

Months	March 2006	April 2006	May 2006	June 2006	July 2006	August 2006	September 2006	October 2006	November 2006	January 2007	February 2007	March 2007
Grass P (mg/g, dry weight) SEM	2.40^d^ ± 0.28	1.74^bc^ ± 0.19	1.17^b^ ± 0.24	0.34^a^ ± 0.09	0.33^a^ ± 0.09	0.35^a^ ± 0.09	1.35^bc^ ± 0.10	1.01^b^ ± 0.09	0.74^b^ ± 0.10	1.10^b^ ± 0.10	1.09^b^ ± 0.10	1.10^b^ ± 0.09
Grass Ca (mg/g, dry weight) SEM	7.53^c^ ± 0.22	7.90^c^ ± 0.20	8.44^c^ ± 0.23	7.29^b^ ± 0.23	7.01^b^ ± 0.30	8.80^c^ ± 0.23	9.01^d^ ± 0.28	7.19^b^ ± 0.23	6.31^a^ ± 0.33	6.45^b^ ± 0.30	6.78^b^ ± 0.30	8.99^d^ ± 0.29
Grass Mg (mg/g, dry weight) SEM	0.47^d^ ± 0.01	0.37^c^ ± 0.01	0.32^b^ ± 0.03	0.34^c^ ± 0.01	0.30^a^ ± 0.03	0.29^a^ ± 0.01	0.29^a^ ± 0.02	0.31^b^ ± 0.01	0.30^b^ ± 0.02	0.35^c^ ± 0.01	0.28^a^ ± 0.01	0.33^c^ ± 0.01
BCS SEM	3.67^a^ ± 0.12	3.57^a^ ± 0.15	3.71^a^ ± 0.15	3.48^a^ ± 0.15	3.52^a^ ± 0.15	3.35^a^ ± 0.20	3.35^a^ ± 0.15	3.45^a^ ± 0.15	3.70^a^ ± 0.15	3.90^a^ ± 0.28	2.96^a^ ± 0.12	3.89^a^ ± 0.28
BM (kg) SEM	377.8^a^ ± 18.3	366.4^a^ ± 18.3	377.1^a^ ± 18.3	360.1^a^ ± 18.3	370.8^a^ ± 18.3	363.4^a^ ± 18.2	362.0^a^ ± 18.2	372.4^a^ ± 20.2	402.1^a^ ± 34.3	444.1^a^ ± 34.3	386.3^a^ ± 32.3	410.3^a^ ± 34.3
Rainfall (mm)	111.2	93.6	9.7	4.2	0	19.3	1	14.1	33.8	60.8	9.7	21.7

^abcd^Means with different letters in the same row are significantly (*P* < 0.05) different

Grass Ca concentrations did not follow the pattern of P. Following 2 months of good rainfall in March 2006 (111.3 mm) and April 2006 (93.6), there were no significant (*P* < 0.05) increases in the concentration of Ca in the grass in April 2006, May 2006, June 2006, or July 2006, and in June 2006 and in July 2006, there were even significant (*P* < 0.05) decreases in grass Ca (7.29 mg/g and 7.01 mg/g dry weight). In August 2007, there was a significant (*P* < 0.05) increase in grass Ca (8.80 mg/g) despite the fact that there was no rainfall in July 2006 (Table 1). The difference in the response of grass P and Ca to the rainfall is seen in the results in June 2006, July 2006, and August 2006. Grass P was significantly (*P* < 0.05) lower in June 2006, July 2006, and August 2006 compared with those months before and after, while grass Ca was significantly (*P* < 0.05) higher in August 2006 compared with June 2006 and July 2006 despite the fact that there was no rainfall in July 2006. In addition, there were no significant differences in grass P during the months of September 2006 through March 2007, but grass Ca showed significant (*P* < 0.05) differences during those same months ranging from a high level of 9.01 mg/g dry weight in September 2006 to a low level of 6.31 mg/g dry weight in November 2006 and then another high level in March 2007 of 8.99 mg/g dry weight even when there were rainfalls of 33.8 mm in November 2006 and 60.8 mm in January 2007. Grass Ca concentrations were not as dependent on rainfall as were grass P concentrations, because there was a significant (*P* < 0.05) increase in grass Ca in August 2006 compared with July 2006 after no rainfall in July 2006, and there was a significant (*P* < 0.05) increase in grass Ca in March 2007 compared with February 2007 despite a very low rainfall (9.7 mm) in February 2007 (Table 1). For animals that depend on grass as their only source of minerals, this has implications for the animal’s ability to maintain a normal Ca:P ratio with much different responses of grass Ca and P to rainfall as seen in August 2006 where there was significantly (*P* < 0.05) less grass P but significantly (*P* < 0.05) more grass Ca.

Grass Mg values in response to rainfall were similar to grass P concentrations except that the grass Mg values responded to the decrease in rainfall in May 2006 faster than did the grass P. Grass P was significantly (*P* < 0.05) less in June 2006, but grass Mg was not significantly lower in May, June, and July 2006, and grass Mg remained significantly lower through September 2006 while grass P was significantly higher in September 2006. This would indicate that the grass was able to increase its P concentration quicker in response to rainfall than it was to increase its Mg concentration. This was also the case in September 2006 through March 2007 when the grass was able to maintain consistent concentrations of P (Table 1), but the grass was not able to maintain consistent values for Mg with significantly higher values in October 2006 (0.31 mg/g) and November 2006 (0.30 mg/g) compared with August 2006 and September 2006, but an even significantly higher value in January 2007 (0.35 mg/g) followed by a significantly lower value in February 2007 (0.28 mg/g) and then a significantly higher value in March 2007 (0.33 mg/g) (Table 1). This has very important implications for the prevention of conditions such as grass tetany in animals maintained only on grass as their only source of Mg.

There were no significant (*P* > 0.05) differences in the BCS or in the BM throughout the experiment period despite the significant (*P* < 0.05) differences seen in P, Ca, and Mg values during the same period in response to the different rainfall patterns. This could be explained in part by the fact that the pastures were overgrazed and so the grass was always in short supply and the animals were never able to consume large amounts of grass over a long period of time and gain weight. What growth of grass there was from the rains was quickly consumed by large numbers of animals communally grazed in the area. Although they were not significant (*P* > 0.05), the BCS of 3.71 and the BM of 377.05 kg were both higher in May 2006 than those in April 2006 (3.57 and 366.38 kg) in response to 111.2 mm rainfall in March 2006 and 93.6 mm in April 2006.

Ruminants are not able to maintain or store large amounts of Mg in the body and rely on a constant source of Mg in the diet for prevention of conditions such as grass tetany (Goff 2008). The results of this research bring attention to the fact that communally grazed animals require mineral supplementation, especially P and Mg for the prevention of mineral deficiencies.
